# Differential changes in life cycle-event phenology provide a window into regional population declines

**DOI:** 10.1098/rsbl.2022.0186

**Published:** 2022-08-31

**Authors:** Hugh J. Hanmer, Philipp H. Boersch-Supan, Robert A. Robinson

**Affiliations:** ^1^ British Trust for Ornithology, The Nunnery, Thetford, Norfolk IP24 2PU, UK; ^2^ Department of Geography, University of Florida, Gainesville, FL 32611, USA

**Keywords:** phenology, moult, annual cycle, breeding, climate change, population change

## Abstract

Climate change affects the phenology of annual life cycle events of organisms, such as reproduction and migration. Shifts in the timing of these events could have important population implications directly, or provide information about the mechanisms driving population trajectories, especially if they differ between life cycle event. We examine if such shifts occur in a declining migratory passerine bird (willow warbler, *Phylloscopus trochilus*), which exhibits latitudinally diverging population trajectories. We find evidence of phenological shifts in breeding initiation, breeding progression and moult that differ across geographic and spring temperature gradients. Moult initiation following warmer springs advances faster in the south than in the north, resulting in proportionally shorter breeding seasons, reflecting higher nest failure rates in the south and in warmer years. Tracking shifts in multiple life cycle events allowed us to identify points of failure in the breeding cycle in regions where the species has negative population trends, thereby demonstrating the utility of phenology analyses for illuminating mechanistic pathways underlying observed population trajectories.

## 1. Introduction

Recent climate change has affected ecological systems in many different ways [1]. Changes in the timing and duration of key life cycle events such as migration and breeding have occurred in birds and other taxa [2–4], which in turn may alter the timing of related ecological processes such as intertrophic relationships or competitive interactions [5]. In particular, if the phenology of events changes at different rates relative to each other, conflict may arise between them, leading to population impacts [6]. Such phenological changes may also not be uniform within a population's range leading to not only differential shifts between life cycle events overall but potentially also to heterogeneity in phenological shifts within different parts of species populations, creating differential drivers of population change.

Alongside breeding and migration, feather replacement (moult) is an energetically costly event in the annual cycle of birds [7,8]. In many species, moult occurs almost immediately following the cessation of breeding and is linked to the regression of gonads [9]. Although both breeding and moult are affected by photoperiod [10,11], increased temperatures have also been implicated in phenological changes in both processes [12,13]. If these life cycle events shift differentially under the same conditions, vital rates such as productivity or survival could be impacted [5,14]. If the timing of breeding advances more, the impact could beneficially lengthen the potential breeding season and increase productivity [15], while a greater advance in timing of moult shortens the potential breeding window and/or increases parent–offspring conflict leading to lower productivity [8,16]. Alternatively, as breeding failure can trigger early moult [7], differential phenological shifts could reflect regional changes in reproductive success [17] due to differential warming in relation to latitude and/or photoperiod [10,11]. Therefore, a better understanding of phenological sensitivities of breeding and post-breeding moult may help us understand the potential implications of climate change in the future.

Willow warblers (*Phylloscopus trochilus*) are an ideal model species for exploring differential phenological shifts. One of Europe's most abundant Afro-Palaearctic breeding migrants, willow warbler populations are in long-term decline across Europe [18] although trends are regionally heterogeneous [19]. Within Great Britain, the population (ssp. *trochilus*) shares a common migratory strategy, but is growing in the north (*ca* 30% increase 2000–2018) and declining in the south (*ca* 35% decline; electronic supplementary material, figure S6) [20,21]. Climate change has been identified as a potential driver of their differing regional population trends, most likely by affecting breeding success [17]. As in many species, the timing of life cycle events differs between the sexes. The female incubates and broods the young alone and, although both parents contribute to provisioning [22], males initiate moult earlier than females [23,24]. Understanding regional variation in phenological shifts in breeding and moult under climate change could help us further understand where in the annual cycle present and future impacts of climate change impact population dynamics in this and other species. We explore the relationship between the timing of two breeding season events (egg laying and incubation) and a post-breeding event (moult) across gradients of both temperature change and latitude as a potential proxy for current and future climate change.

We hypothesize that the timing of each life cycle event will advance both in warmer years and at more southerly latitudes and quantify the extent to which these advances may differ between events. Further, given the regionally divergent population trajectories, we expect any differences to be greater towards the south, where population trends are more negative.

## 2. Methods

We combine observations of three life cycle events (egg laying, brood patch re-feathering, primary feather moult) from two large-scale citizen science schemes, the UK Nest Record Scheme [25] and the British and Irish Ringing Scheme [26], collected from 2000 to 2018. Nest recording and ringing occur at volunteer-selected sites, with good coverage of Great Britain north of 51°N and south of 57°N and substantial spatial overlap between the schemes (electronic supplementary material, figures S4 and S5).

### (a) Timing of breeding

We determined the laying date of the first egg (hereafter clutch initiation) from repeated observations of nest contents for 1014 nests where this could be estimated to ± 10 days [25]. Expected clutch completion dates were calculated by adding the species' mean clutch size (five eggs, based on laying one egg per day [22]) to the clutch initiation date.

Brood patch status was recorded in 5268 captured females on a six-point scale where 0 represents brood patch absence, 3 a fully engorged patch and 5 a re-feathering patch [27]. We used scores of 4–5 as an indicator of the start of brood patch re-feathering, which occurs at the end of incubation or the loss of a clutch [28,29].

### (b) Timing of moult

We analysed 14 229 moult records (7175 female and 7054 male) from adult willow warblers captured after 30 April (day 120) each year. The presence of a brood patch (females) or cloacal protuberance (males) was used to assign sex, with a binary classifier using wing length [30] used for birds lacking sex information (electronic supplementary material).

Primary feather tract moult status was recorded as a categorical variable (not started, in progress, completed) with progression of individual feather growth scored using a six-point scale where 0 is an old unmoulted feather and 5 a new fully grown feather [31]. The sum of the 10 primary feather scores for one wing (a scale of 0–50) was converted into the proportion of new primary feather mass grown using feather-specific masses [23].

### (c) Climatic variables

Gridded annual spring (March–May) mean temperature observations were sourced from HadUK-Grid [32] for a 5 km buffer area around the locations of each capture and nest record and converted to local and annual mean spring temperature anomalies (*T_s_*; [33]), i.e. annual departures from the location-specific 2000–2018 mean spring temperature (in °C).

### (d) Statistical analysis

#### (i) Clutch initiation phenology

We fitted linear mixed-effects models using R package lme4 (1.1-27.1; [34]) to estimate clutch initiation with respect to *T_s_* and latitude (as a continuous variable, centred and scaled by 1 s.d.) and their interaction, with year included as a random factor to account for any unevenness in sampling effort through time.

#### (ii) Brood patch phenology

We fitted mixed-effects probit regression models (electronic supplement material; [35]) using R package glmmTMB (1.1.2.3; [36]) to estimate the onset of brood patch re-feathering with respect to *T_s_* and latitude (as above) and their interaction, with year as a random factor.

#### (iii) Moult phenology

Primary moult initiation date, its population variance and moult duration were estimated using moult phenology models [37] in R package moult (2.2.0; [38]). As willow warblers commence migration soon after moult completion, we used type 5 models which include observations from pre-moult and active moult only [38,39]. Moult duration and initiation were considered in respect to *T_s_* and latitude (treated as above) and their interaction with sex was included as an additive covariate for the latter and also for the variance in population moult initiation date.

Linear predictor structures for the models of each life cycle event were selected using AIC model selection ([40]; electronic supplementary material, tables S1, S3 and S5).

## 3. Results

**Figure 1.**
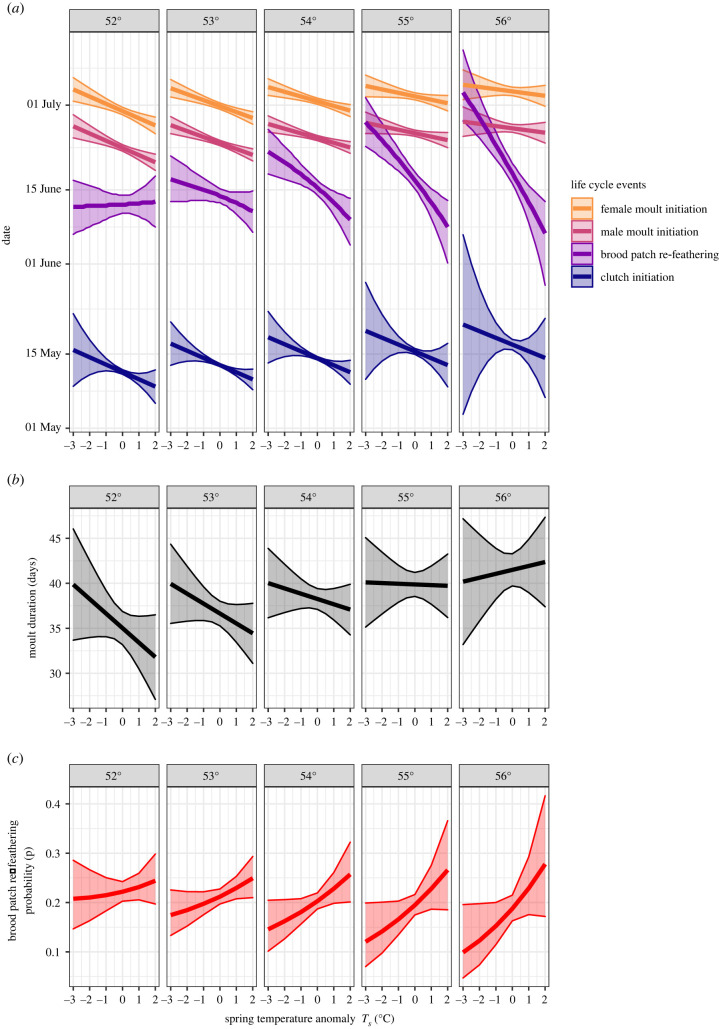
Willow warbler (*a*) breeding and moult phenology, (*b*) moult duration and (*c*) brood patch re-feathering probability at clutch completion with respect to spring temperature anomaly size (*T*_s_), latitude and their interaction. Plotted lines show conditional effect estimates at five locations across the studied latitudinal range with 95% CIs based on the models in tables 1 and 2.

Breeding started (clutch initiation; −1.3 days/°C) and ended (brood patch re-feathering; −2.6 days/°C) earlier, and birds moulted earlier (−0.9 days/°C) and more quickly (−0.6 days/°C; all effects given at 54°N; tables 1 and 2, figure 1) in warmer years. Clutch initiation (1.3 days/°N), brood patch re-feathering (1.5 days/°N) and moult initiation (0.9 days/°N) all become later and moult duration was longer (1.6 days/°N; all effects at *T_S_* = 0°C) at higher latitudes. Males initiated moult 7 days earlier than females and were more synchronous in their start date (s.d. of start date 3.4 days shorter; table 1, figure 1*a*). Model selection favoured models with a *T_s_*–latitude interaction (ΔAIC greater than or equal to 4; electronic supplementary material, tables S1, S3 and S5), the size and sign of which differed between the life cycle events (figure 1). It had little effect on clutch initiation but a pronounced effect on the other life cycle events. In the coolest springs, moult initiation showed little phenological sensitivity across latitudes, but in the warmest springs southerly locations experienced a more pronounced shift in moult initiation compared to the north (figure 1*a*). Moult duration estimates were more uncertain overall and similar across latitudes in cool springs but shortened considerably in the south in warm springs compared to the north (figure 1*b*). The mean timing of brood patch re-feathering showed the opposite trend, with populations in the south exhibiting little phenological sensitivity to temperature, but more northerly populations showing an increasing negative effect with *T_s_* (figure 1*a*, table 2). This led to the probability of brood patch re-feathering in an individual increasing more markedly at the time of clutch completion in warmer years in the north compared to the south (figure 1*c*). As the breeding season progressed, the effect of *T_s_* on brood patch re-feathering reversed in the south, but not in the north (electronic supplementary material, figure S7), resulting in a drawn out period of brood patch re-feathering across southern populations in cold springs, with some individuals re-feathering earlier than in the north, but others re-feathering later.

**Table 1.** Parameter estimates (mean and 95% confidence interval from a linear mixed-effects model and an Underhill–Zucchini moult model) for willow warbler life cycle events. Asterisk (*) indicates an interaction.

life cycle event	intercept (female)	*T_s_* (°C)	latitude (standardized)	*T_s_* * latitude	sex (Male)
clutch initiation date	133.6	−1.3	2.2	0.1	
	(132.8–134.3)	(−2.5 – −0.5)	(1.4–3.0)	(−1.0–1.1)	
moult duration	37.3	2.7	−0.9	0.9	
	(36.1–38.6)	(1.5–3.9)	(−2.2–0.4)	(−0.6–2.3)	
moult initiation date	182.2	−1.0	1.6	0.4	−7.0
	(181.6–183.8)	(−1.5 – −0.5)	(1.2–2.0)	(−0.1–0.9)	(−7.7 – −6.3)
standard deviation in moult initiation date	14.0				−3.4
	(10.6–17.5)				(−6.0–−0.9)

**Table 2.** Parameter estimates (mean risk ratio and 95% confidence interval from mixed-effects probit model) for willow warbler life cycle events. Asterisk (*) indicates an interaction.

	intercept	ordinal day	latitude (standardized)	*T_s_*(°C)	ordinal day * latitude	ordinal day * *T_s_*	latitude * *T_s_*	ordinal day * latitude * *T_s_*
brood patch re-feathering probability	0.0003 (0.0002–0.0004)	1.051 (1.048–1.053)	0.675 (0.514–0.888)	1.791 (1.096–2.926)	1.002 (1.000–1.004)	0.997 (0.994–1.000)	0.918 (0.602–1.399)	1.001 (0.999–1.003)

## 4. Discussion

Our results show that willow warblers in the south moult earlier and faster in warm springs compared to cooler springs and more northerly latitudes, whereas the relationship between first egg date and local spring temperature remains much the same across all latitudes. Brood patch re-feathering occurs earlier in warmer springs with the effect being most marked in the north. However, with the exception of the warmest springs, overall levels of brood patch re-feathering were higher in the south early in the breeding season, when they likely reflected early brood failures or smaller brood sizes [41]. Towards the end of the breeding season, the effect of temperature on brood patch re-feathering was reversed (electronic supplementary material, figure S7) indicating an overall later completion of incubation in cold springs, likely a result of incubating replacement and/or second broods. There is a similar latitudinal gradient in population trends, with those in northern Britain (Scotland) increasing (by 25%), while those in the south (England) have decreased by 45% [21]. The differential shifts in life cycle-event phenology detected in this study provide a window into the mechanisms underlying the observed differences in regional population trajectories. In particular, we show that both brood patch re-feathering and moult occur earlier in regions where the species has negative population trends, pinpointing a source of poor breeding success early in the breeding season with increasing warming.

There is abundant evidence for advances in breeding with increased spring temperatures under climate change in many species, including willow warbler [12]. Warmer temperatures have been found to advance gonad regression and moult initiation experimentally, which could explain earlier cessation of breeding [9]. For willow warbler, shorter breeding seasons as a result of temperature-induced earlier moult would reduce the possibility of replacement (and second) broods and the extent of post-fledging care [13,16] and so contribute to a decline in reproductive success in the south. Changes in breeding season length with climate change have been identified in a number of temperate/boreal bird species, with warming correlated to shorter breeding seasons in single-brooded species, potentially reducing productivity output [15,42].

Alternatively, differential changes in moult initiation following warmer springs could be a direct consequence of changes in the reproductive phase. Declines in southern willow warbler populations have been linked to lower productivity in some years, possibly due to increased rates of nest failure [17]. If nest failure is more likely in warmer springs, then adults may abandon breeding and start moulting earlier. Our findings indicate that the earlier start to the breeding season in warmer years has reduced potential moult–breeding conflict in northern populations, but similar shifts in the south have not resulted in more positive population trends. This is presumably because warming is also associated with a higher proportion of early nest failures (indicated by brood patch re-feathering) or smaller broods [41] in the south, and subsequently earlier primary moult. Given experimental evidence of earlier moult in warmer conditions [9], it is plausible these effects act together to produce the observed regional differential shift in moult phenology, which may become even more pronounced with further climate change. This is especially important given we find evidence that warmer northern springs shift brood patch re-feathering even earlier suggesting recent northern population gains may be reversed under further warming.

The consequences of the more uncertain concurrent differential shift observed in willow warbler moult duration, and by extension moult intensity, under the same conditions as moult initiation remain unclear. Warmer ambient temperatures and consequently reduced metabolic costs may mean more energy is available to increase primary moult intensity [43,44], thus shortening the period of impaired flight and potentially increasing pre-departure survival [7]. However, faster moult may also reduce feather quality and thus negatively impact survival during the southward migration [45,46]. No regional differences in adult survival have been identified or linked to the observed regional population trends [17], so the impact of this apparent change in moult duration appears minimal at present.

Although studies of relative change in phenology between species are increasingly common, we find that recording and analysing potentially differential phenological changes within a species across multiple life cycle events may shed light on the drivers of population trends. Under climate change such differential shifts may have important and overlooked consequences not only in birds but also across other taxa (e.g. [47]).

## Data Availability

Gridded climatic variables are available from: https://catalogue.ceda.ac.uk/uuid/4dc8450d889a491ebb20e724debe2dfb. Bird records and processed climate are available from the Dryad Digital Repository: https://doi.org/10.5061/dryad.v6wwpzgzv [48]. Electronic supplementary material is available online [49].
